# An optimized protocol for expression and purification of monomeric full-length BAX protein for functional interrogations

**DOI:** 10.3389/fcell.2023.1322816

**Published:** 2023-12-08

**Authors:** Yiyang Chen, Jesse D. Gelles, Jarvier N. Mohammed, Jerry Edward Chipuk

**Affiliations:** ^1^ Laboratory of Mitochondrial Biology in Human Health and Disease, Icahn School of Medicine at Mount Sinai, New York,NY, United States; ^2^ Department of Oncological Sciences, Icahn School of Medicine at Mount Sinai, New York, NY, United States; ^3^ The Tisch Cancer Institute, Icahn School of Medicine at Mount Sinai, New York, NY, United States; ^4^ The Graduate School of Biomedical Sciences, Icahn School of Medicine at Mount Sinai, New York, NY, United States; ^5^ Department of Dermatology, Icahn School of Medicine at Mount Sinai, New York, NY, United States; ^6^ The Diabetes, Obesity and Metabolism Institute, Icahn School of Medicine at Mount Sinai, New York, NY, United States

**Keywords:** apoptosis, BAX, BCL-2 family, mitochondria, mitochondrial outer membrane permeabilization, recombinant protein production

## Abstract

Diverse developmental signals and pro-death stresses converge on the regulation of the mitochondrial pathway of apoptosis. BAX, a proapoptotic BCL-2 effector, directly forms proteolipid pores in the outer mitochondrial membrane to activate the mitochondrial pathway of apoptosis. BAX is a viable pharmacological target for various human diseases, and increasing efforts have been made to study the molecular regulation of BAX while identifying small molecules selectively targeting BAX. However, generating large quantities of monomeric and functionally competent BAX has been challenging due to its aggregation-prone nature. Additionally, there is a lack of detailed and instructional protocols available for investigators who are not already familiar with recombinant BAX production. Here, we present a comprehensive protocol for expressing, purifying, and storing functional monomeric recombinant BAX protein. We use an intein-chitin binding domain-tagged BAX-expressing construct and employ a two-step chromatography strategy to capture and purify BAX. We also provide examples of standard assays to observe BAX activation, and highlight the best practices for handling and storing BAX to effectively preserve its quality, shelf life, and function.

## Introduction

Apoptosis is a fundamental physiological process essential for tissue development and homeostasis. The BCL-2 family of proteins governs the mitochondrial pathway of apoptosis by regulating mitochondrial outer membrane permeabilization (MOMP), an event considered the “point of no return” and a cellular commitment to apoptotic death. Following MOMP, several apoptogenic factors, such as cytochrome *c*, are released from the mitochondrial intermembrane space into the cytosol, which triggers the caspase cascade for cellular dismantling.

BAX, a proapoptotic BCL-2 effector protein, directly mediates MOMP by forming proteolipid pores on the outer mitochondrial membrane (OMM). Nascent BAX exists as a soluble monomer, with its ɑ9 helix occupying the BH3- and C-terminal (BC) groove (Suzuki et al., 2000). In response to apoptotic stimuli, BAX is activated by a subset of BCL-2 family proteins (“direct activators,” such as BIM) (Kuwana et al., 2002; Gavathiotis et al., 2008). Upon activation, BAX undergoes a series of conformational changes, including mobilization of the ɑ9 helix from the BC groove, which facilitates the translocation of BAX to mitochondria; oligomerization; and subsequent pore formation in the OMM (Gross et al., 1998; Antonsson et al., 2000). Given the central role of BAX in initiating apoptosis, there have been an increasing number of efforts to study the structure–function relationship and molecular regulation of BAX, which has led to the discovery of several regulatory sites (Gavathiotis et al., 2008; Czabotar et al., 2013; Barclay et al., 2015). Furthermore, several studies have identified or developed small molecules capable of directly targeting BAX and modulating its function (Gavathiotis et al., 2012; Niu et al., 2017; Pritz et al., 2017; Reyna et al., 2017; Garner et al., 2019).

Several multi-domain BCL-2 family proteins are membrane-associated, thereby introducing technical complications in the generation of their full-length recombinant forms. By contrast, BAX uniquely exists as a soluble cytosolic protein in its inactive state, simplifying the process of generating recombinant protein and representing a biologically relevant state for studies assessing the structure–function relationship. Various biophysical and biochemical techniques commonly utilized in BAX research (e.g., membrane permeabilization assays, fluorescence polarization, ITC, and NMR) require large quantities of highly pure and functionally competent BAX protein. Therefore, reliably generating high-quality recombinant BAX is indispensable for BAX research. However, due to the aggregation-prone nature of BAX, the process of generating, handling, and storing recombinant BAX is a challenge.

**FIGURE 1:**
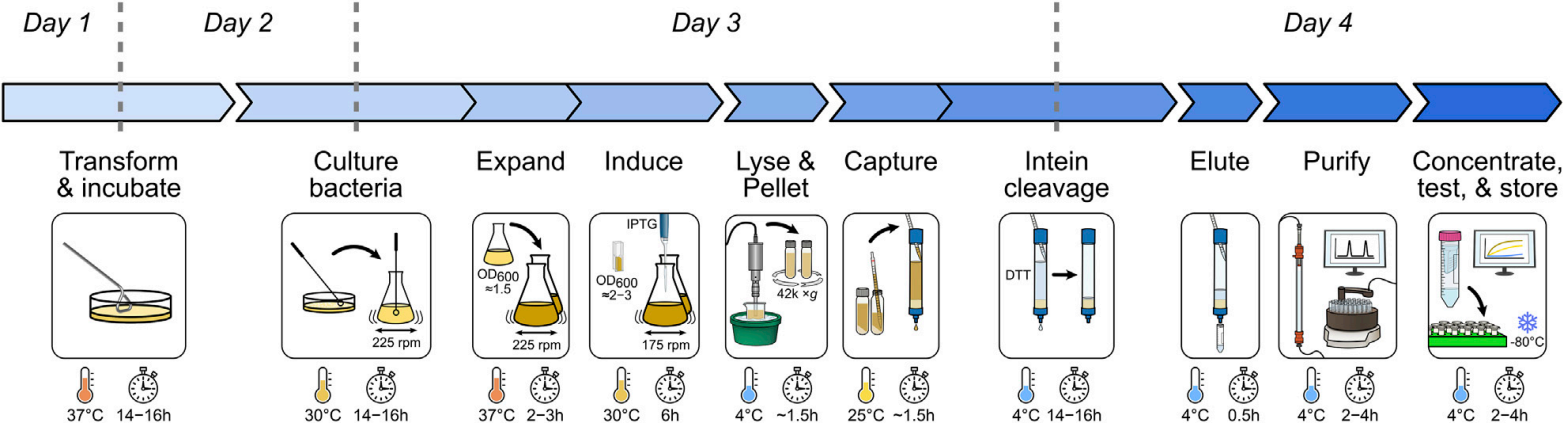
Overview of the recombinant BAX purification workflow. Major expression and purification steps are visualized with essential parameters marked.

Here, we present an optimized protocol for the generation of a high-purity, monomeric, full-length recombinant human BAX. We use an intein–chitin binding domain (CBD)-tagged BAX construct and employ a two-step chromatography strategy to capture and purify monomeric BAX (Figure 1). To aid researchers in validating the functionality of recombinant BAX, we demonstrate a series of gold standard assays to ensure that BAX is highly responsive to various BAX activators. Furthermore, we optimized the storage conditions to effectively preserve the high quality of BAX, ensuring consistency across a spectrum of experiments. Taken together, BAX generated by using this protocol is properly folded, monomeric, functionally competent, stable, and devoid of exogenous residues or modifications in its sequence. Collectively, this comprehensive protocol is beneficial for investigators who are not familiar with recombinant BAX production and serves as a useful resource for experienced investigators to compare best practices.

## Materials and equipment

### Laboratory equipment

• High-speed centrifuge (e.g., Avanti J-E Series; Beckman Coulter)
• Incubator shaker capable of holding a 2-L flask (e.g., Innova 44; New Brunswick Scientific)
• Liquid chromatography systems kept at 4°C (e.g., ÄKTA pure™ 25 L1; Cytiva)
• Microfuge-tube centrifuge (e.g., Legend Micro 21R; Thermo Scientific)
• Microplate reader capable of absorbance and fluorescence (e.g., Synergy H1; BioTek)
• Probe sonicator (e.g., Dismembrator 505; Fisher Scientific)
• Protein electrophoresis and Western blot sets (e.g., Criterion Vertical Electrophoresis Cell; Bio-Rad)
• Spectrophotometer capable of cuvette readings (e.g., Ultraspec 7000; GE)
• Size exclusion column (e.g., HiLoad 16/600 Superdex 200 pg column, Cytiva)
• Table-top swinging bucket centrifuge with 50-mL tube adapters (e.g., Legend XTR Centrifuge; Thermo Scientific)
• ThermoMixer (e.g., ThermoMixer; Eppendorf)

### Consumables and labware

• 1.5-mL Eppendorf tubes (Cat. no. 4036-3204, USA Scientific)
• 1.5-mL semi-micro cuvettes (Cat. no. 14955127, Thermo Scientific)
• 2-L culture flask (Cat. no. CLS431256, Sigma-Aldrich)
• 5-mL syringe with a luer-lock tip (Cat. no. 309646, BD)
• 50-mL conical centrifuge tubes (Cat. no. SC-200251, Santa Cruz Biotechnology)
• 96-well plate, flat bottom, black polystyrene (Cat. no. 3915, Corning)
• 96-well clear plates
• Amicon^®^ Ultra-4 Centrifugal Filters, 10 kDa MWCO (Cat. no. UFC801024, Sigma-Aldrich)
• Gravity chromatography column (25 mm × 200 mm) (Cat. no. SC-205557, Santa Cruz Biotechnology)
• Oak Ridge centrifuge tube (Cat. no. 3119-0050, Thermo Scientific)
• Petri dish, 100 × 15 mm (Cat. no. FB0875712, Fisher Scientific)
• Serological pipettes of various sizes (Globe Scientific)

### Reagents

• Carbenicillin (Cat. no. 20871, Cayman Chemical)
• Chitin resin (Cat. no. S6651L, New England Biolabs)
• Dithiothreitol (DTT) (Cat. no. DTT10, Gold Biotechnology)
• BL21 Star (DE3) cells (Cat. no. C601003, Thermo Fisher Scientific)
• GelCode™ Blue Stain Reagent (Cat. no. 24592, Thermo Scientific)
• Gel filtration molecular weight standard (Cat. no. 1511901, Bio-Rad)
• Glycerol (Cat. no. BP229-1, Fisher Scientific)
• 4-(2-hydroxyethyl)-1-piperazineethanesulfonic acid (HEPES) (Cat. no. A14777.30, Thermo Scientific)
• Isopropyl-β-D-thiogalactoside (IPTG) (Cat. no. I2481C, Gold Biotechnology)
• Luria–Bertani (LB) agar, Miller’s (Cat. no. DF0445-17-4, Fisher Scientific)
• LB broth (Miller’s) (Cat. no. BD 244610, Fisher Scientific)
• 1× Phosphate-buffered saline (PBS) [pH 7.4] (Cat. No. 10010-023, Gibco)
• Pierce™ Protease Inhibitor cocktail (Cat. no. A32965, Thermo Fisher Scientific)
• Potassium phosphate dibasic (K_2_HPO_4_) (Cat. No. BP363-500, Fisher Scientific)
• Sodium chloride (NaCl) (Cat. no. 447300050, Thermo Scientific)
• Sodium phosphate monobasic (NaH_2_PO_4_) (Cat. no. BP329-1, Fisher Scientific)
• Terrific Broth (TB) medium (Cat. no. DF0438-17, Fisher Scientific)

### Buffer formulations

• 2.5× TB broth (125 g of dehydrated TB culture medium/L; 10 mL glycerol/L)
• Gel filtration buffer [150 mM NaCl; 10 mM HEPES (pH 7.4)]
• Lysis buffer (500 mM NaCl, 50 mM K_2_HPO_4_, and 50 mM NaH_2_PO_4_), freshly supplemented with a Pierce^TM^ Protease Inhibitor cocktail tablet according to the manufacturer’s instructions

### Kit

• Pierce™ BCA Protein Assay Kits (Cat. no. 23225, Thermo Scientific)

### Antibodies for Western blotting

• BAX antibody (2D2) (Cat. no. SC-20067, Santa Cruz Biotechnology)
• Cytochrome *c* antibody (Cat. no. 54205-RBM6-P1, Thermo Scientific)
• m-IgGκ BP-HRP (Cat. no. sc-516102, Santa Cruz Biotechnology)

### Plasmids

• pTYB1–BAX [100 ng/μL] was used for expression of intein-CBD-tagged BAX. BAX cDNA was subcloned into the NdeI/SapI cloning site of the pTYB1 vector, which results in a C-terminal tag with no exogenous residues (Suzuki et al., 2000).

### Materials for BAX validation assays (optional)

• (3-cholamidopropyl)dimethylammonio)-1-propanesulfonate (CHAPS) (Cat. no. C-080, Gold Biotechnology)
• 8-aminonapthalene-1,3,6-trisulfonic acid (ANTS) (Cat. no. A350, Thermo Fisher Scientific)
• BSA fraction V (Cat. no. 12-660-9100GM, Fisher Scientific)
• Cardiolipin (18:1) (Cat. no. 710335C, Avanti Polar Lipids)
• Dodecylphosphocholine (DDPC) (Cat. no. 25629, Cayman Chemical)
• Brain phosphatidylserine (porcine) (Cat. no. 840032C, Avanti Polar Lipids)
• Ethylenediaminetetraacetic acid (EDTA) (Cat. no. 798681-100G, Sigma-Aldrich)
• Ethylene glycol-bis(β-aminoethyl ether)-*N*,*N*,*N*′,*N*′-tetraacetic acid (EGTA) (Cat. no. E0396, Sigma-Aldrich)
• Egg phosphatidylcholine (chicken) (Cat. no. 840051C, Avanti Polar Lipids)
• Egg phosphatidylethanolamine (chicken) (Cat. no. 840021C, Avanti Polar Lipids)
• Human BID-BH3, peptide (Cat. no. AS-61711, AnaSpec)
• Human BIM-BH3, peptide IV (Cat. no. AS-62279, AnaSpec)
• Liver phosphatidylinositol (bovine) (Cat. no. 840042C, Avanti Polar Lipids)
• p-Xylene-bis-pyridinium bromide (DPX) (Cat. no. X1525, Thermo Fisher Scientific)
• Recombinant human BID (caspase-8-cleaved) (Cat. no. 882-B8, R&D Systems)
• Sucrose (Cat. no. S0389, Sigma-Aldrich)
• Trehalose (Cat. no. 1673715, Sigma-Aldrich)

### Buffer formulations for BAX validation assays (optional)

• Large unilamellar vesicle (LUV) buffer [200 mM KCl, 5 mM MgCl_2_, 0.2 mM EDTA, and 10 mM HEPES (pH 7.4)]
• Trehalose isolation buffer (TIB) [200 mM trehalose, 68 mM sucrose, 10 mM HEPES (pH 7.4), 10 mM KCl, 1 mM EDTA, 1 mM EGTA, and 0.1% BSA Fraction V], freshly supplemented with a Pierce^TM^ Protease Inhibitor cocktail tablet according to the manufacturer’s instructions.

## Methods

### Reagents and buffer preparations

Prepare 100 mg/mL carbenicillin (×1,000) stock (6 mL) by dissolving carbenicillin powder in distilled H_2_O (dH_2_O). Then, prepare 1 M IPTG (4 mL) stock by dissolving IPTG powder in dH_2_O and store carbenicillin and IPTG stocks at −20°C and thaw as needed for immediate use.

Prepare 1× LB broth (1 L) by combining 25 g LB powder in 1 L of dH_2_O and thoroughly mix using a magnetic stir bar. Prepare 2.5× TB broth (4 L) by combining 500 g TB powder in 4 L dH_2_O, supplemented with 40 mL glycerol, and thoroughly mix using a magnetic stir bar. Sterilize both LB and TB stocks by using an autoclave and store at 4°C to prevent contamination. Prior to culturing bacteria, freshly supplement the LB and TB with carbenicillin to a final concentration of 100 μg/mL (1:1,000-fold dilution).

Prepare 1× LB agar media (1 L) by combining 40 g of LB agar powder in 1 L dH_2_O and thoroughly mix by applying heat using a magnetic stir bar. Heat the agar mixture to boiling temperature and maintain the temperature for 1 min to completely dissolve the agar. Sterilize the LB agar stock by using an autoclave and cool it down to 45°C–50°C. Supplement the LB agar stock with carbenicillin to a final concentration of 100 μg/mL and pour 20 mL into 10-cm Petri dishes. Once the agar solidifies, store the LB agar plates at 4°C to prevent contamination.

Prepare the lysis buffer (500 mL) by combining 83.3 mL 3 M NaCl, 12.5 mL 2 M K_2_HPO_4_, and 12.5 mL 2 M NaH_2_PO_4_ into 391.7 mL dH_2_O. Prepare the gel filtration buffer (1 L) by combining 50 mL 3 M NaCl and 10 mL 1 M HEPES [pH 7.4] in 940 mL dH_2_O. Critical: BAX can aggregate in the presence of detergent, and common glassware can be contaminated with residual detergent from glassware cleaning. So the best practice is to designate a set of glassware, specifically used for preparing the lysis buffer and gel filtration buffer, and avoid cleaning it with detergents.

### Transform BL21 Star^TM^ (DE3) bacterial cells with the pTYB1–BAX expression vector

Day 1: Thaw 12.5 μL BL21 cells on ice for 10 min before adding 50 ng of the pTYB1-BAX construct. Gently flick the tube several times to mix and incubate on ice for 10 min. Apply heat shock to the BL21 cells at 42°C for 45 s (using a water bath or heating block) and then immediately return the tube to ice for 2 min. Add 750 μL of LB broth to the tube and incubate the culture with agitation in a shaking incubator set to 220 rpm at 37°C for 45 min (note: for best agitation, position the tube at an angle or perpendicular to the direction of the shaker). While the bacteria are incubating, place a prepared LB agar plate supplemented with 100 μg/mL carbenicillin in a 37°C incubator to warm the plate and eliminate condensation. Pellet the cells by centrifuging at 1,500 × *g* for 2 min in a table-top centrifuge. Remove 700 μL of the supernatant using a pipette and resuspend the pellet in the remaining 50 μL of media. Pipette the BL21 cell suspension onto the prewarmed LB agar plate and spread the suspension evenly throughout the plate (using a sterile glass spreader, glass beads, pipette tip, or similar equipment). Incubate the LB agar plate at 37°C for 14–16 h. Note: this incubation step is commonly performed overnight and serves as a breakpoint in the protocol.

### Expand BL21 (DE3) culture and induce the expression of recombinant BAX

Day 2: Remove the culture plate from the incubator and inspect for colony growth. Keep the plate at 25°C until ready to proceed with the next step. Pick a single bacterial colony from the LB agar plate and inoculate into 400 mL of 1× LB freshly supplemented with 100 μg/mL carbenicillin in a 2-L flask. Expand this starter culture in a shaking incubator set to 225 rpm at 30°C for 14–16 h. Note: typically, the plate is removed from the incubator in the morning and a colony is picked several hours later so that the incubation of the starter culture occurs overnight and aligns with the day schedule.

**FIGURE 2:**
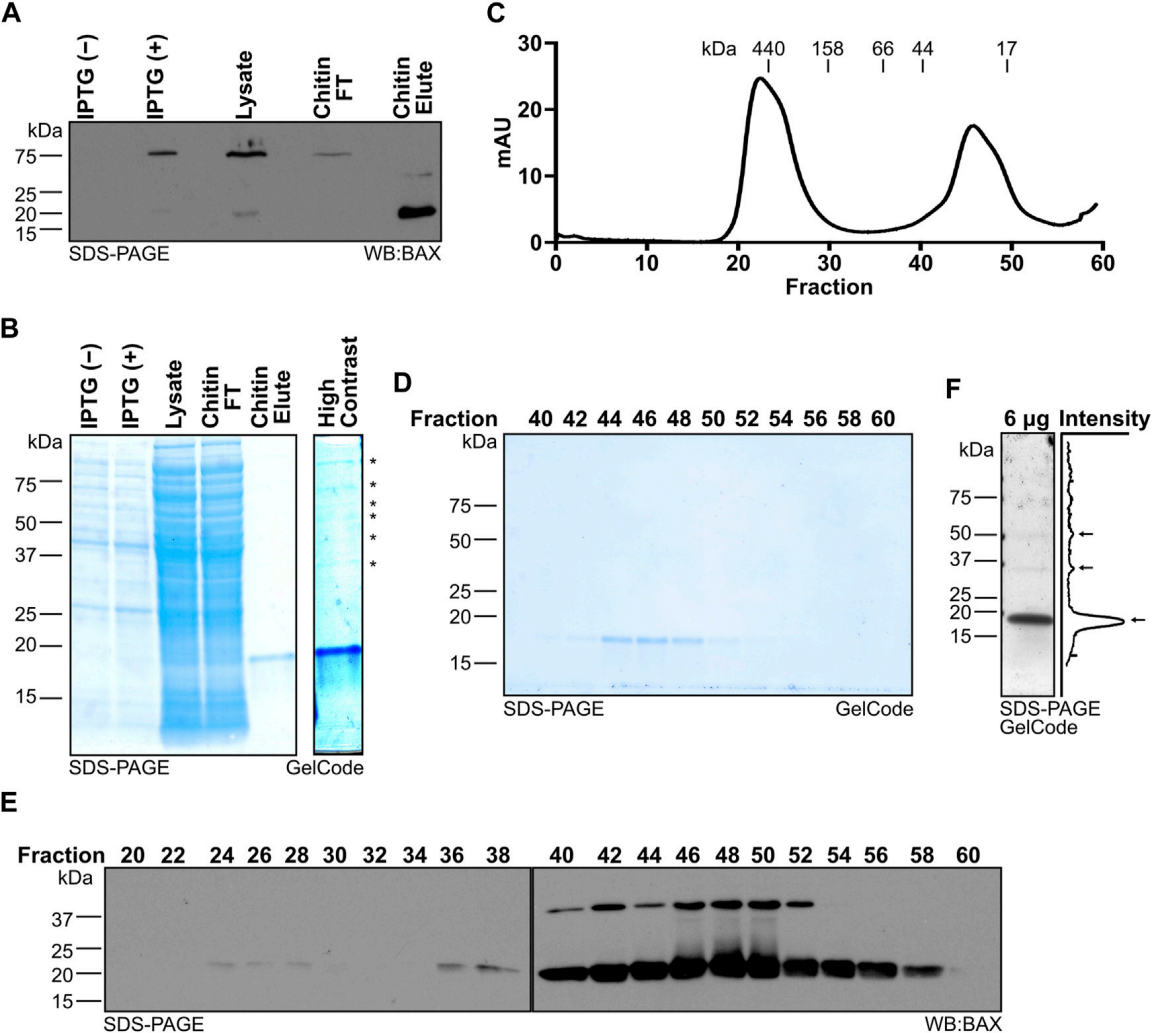
Systematic evaluation of BAX protein expression and purification. **(A)** Western blot detection of BAX for the samples collected throughout protein generation, which reveals the intein-CBD tagged BAX expression induced by IPTG, intein-CBD tagged BAX captured by the chitin column, and BAX release by the DTT-induced intein cleavage. **(B)** Left: protein staining of the samples collected throughout protein generation as in **(A)**; right: high contrast of the chitin eluate, which displays the presence of high molecular weight proteins noted with an asterisk. **(C)** 280-nm absorbance values of the sample exiting the HiLoad 16/600 Superdex 200 pg column. Molecular weights were determined by the gel filtration chromatography standard. **(D)** Protein staining of fractions from **(C)**. **(E)** Western blot detection of BAX for the fractions from **(C**). **(F)** Left: protein staining of 6 μg of the BAX stock; right: quantification of pixel intensity. Pixel intensity was analyzed using Plot Profile in ImageJ/Fiji and averaged across the width of the image. Monomeric and multimeric BAX are marked with arrows.

Day 3: In the morning, take 1 mL of the bacterial culture and measure the optical density at 600 nm (OD_600_) by using a spectrophotometer with 1 mL of dH_2_O as the blank solution. Continue to grow the starter culture until reaching an OD_600_ value of approximately 1.5. Supplement the 400 mL of the bacterial culture with 800 mL of 2.5× TB broth and 800 mL distilled H_2_O (dH_2_O), split it evenly into two 2-L flasks, and incubate them at 37°C with shaking at 225 rpm to further expand the culture. For increased recombinant protein yield, using 2–4 L of the TB culture is advised. Measure the OD_600_ each hour to monitor the population growth by removing 200 μL of the culture and diluting it with 800 μL dH_2_O in a cuvette (5-fold dilution). Use 1 mL of dH_2_O as a blank solution and adjust the OD_600_ value by the dilution factor (i.e., multiply by 5). Grow the culture to a target OD_600_ value of 2–3, which typically is achieved in 2–3 h. Note: it is strongly recommended that the investigators reserve a 100-μL aliquot of the confluent culture as a negative control before IPTG-induction to assess the quality of BAX expression [“IPTG (−);” Figure 2A].

Once the culture is at the target OD_600_, induce the expression of recombinant BAX by supplementing 1 mM IPTG (e.g., 1 mL of 1 M IPTG per liter of TB culture). Continue to incubate the bacterial culture in a shaking incubator set to 175 rpm at 30°C for 6 h. Note: it is strongly recommended that the investigators reserve a 100-μL aliquot of the culture after the 6 h induction to assess the quality of BAX expression [“IPTG (+);” Figure 2A].

### Pellet and lyse BL21 (DE3) cells

Pellet the BL21 (DE3) cells in 1-L canisters by centrifuging at 4,000 × *g*, at 4°C for 15 min in a high-speed centrifuge. Decant the supernatant, and wash the bacterial pellet in the canister by resuspending with 25 mL of cold 1× PBS. Transfer the cell suspension to a 50-mL centrifuge tube, and pellet the cells by centrifuging at 4,000 × *g*, at 4°C for 45 min. Decant the PBS after centrifugation. Note: the cell pellet may be flash-frozen in liquid nitrogen for future lysis; however, this may lead to reduced protein yield due to the additional freeze-thaw cycle.

Resuspend the BL21 cell pellet in 35 mL of the lysis buffer per 5 mL of the pellet volume and ensure that the cells are thoroughly resuspended through vigorous vortexing for optimal lysis efficiency. Combine the suspensions in a beaker placed on ice and continue to resuspend for 15 min using a magnetic stir bar. Freshly supplement the cell suspension with Pierce™ Protease Inhibitor cocktail following the manufacturer’s instructions. While keeping the beaker on ice, use a probe sonicator to lyse the cells using eight cycles of the following program: run time at 2 min; PULSAR on at 15 s and PULSAR off at 2 min per cycle. Note: once the cells are lysed, it is strongly recommended that the investigators reserve a 100-μL aliquot of the cell lysate to evaluate the binding efficiency of the recombinant BAX protein to the chitin resin (“Lysate;” Figure 2A).

### Capture the recombinant BAX using a chitin affinity column

Recombinant BAX expressed by the pTYB1 vector has an intein-CBD tag at its C-terminus, suitable for affinity chromatography using chitin beads. A thiol-induced self-cleavage of intein releases BAX from the chitin beads, resulting in an eluate containing full-length BAX with no exogenous residues (Chong et al., 1998).

Transfer the cell lysate into 40-mL Oak Ridge tubes and centrifuge in the high-speed centrifuge at 42,000 × *g*, at 4°C for 1 h to pellet the cell debris. During the centrifugation, prepare the chitin column for capturing BAX. Gently swirl the chitin resin in its container to resuspend it. For each liter of the TB culture, add 20 mL of the chitin resin slurry (equivalent to 10 mL of the chitin-resin bed volume) into a gravity column and allow the resin to settle. Equilibrate the chitin column by washing twice with three bed volumes of dH_2_O followed by washing twice with three bed volumes of the prechilled lysis buffer. Seal the chitin resin column when the lysis buffer level is approximately 0.5 cm higher than the resin layer. Preparing the column and chitin capture can be performed on the bench (∼25°C).

Once the centrifugation is finished, load the supernatant directly onto the chitin column by using a serological pipette, and then unseal the chitin column to allow the supernatant to flow through the chitin resin. Collect the flow-through until the supernatant level is approximately 0.5 cm higher than the resin layer and then cap the column. Reapply the collected flow-through back onto the column and allow it to pass through again for additional BAX capture. Repeat once again for a total of three passes of the supernatant through the column and cap after the final pass. Note: after the final capture, it is strongly recommended that the investigators reserve a 100-μL aliquot of flow-through (FT) to evaluate the binding efficiency of the recombinant BAX protein to the chitin resin (“Chitin FT;” Figure 2A).

Wash the chitin column twice with three bed volumes of the prechilled lysis buffer, followed by equilibrating the column by washing three times with 15 mL lysis buffer, freshly supplemented with 50 mM DTT. During the final wash, seal the chitin column when the lysis buffer level is approximately 0.5 cm higher than the resin layer. Incubate the chitin resin at 4°C for a minimum of 16 h for on-column, DTT-induced cleavage of the intein tag and release of BAX from the chitin resin. Note: this step is commonly performed overnight and serves as a breakpoint in the protocol.

At the end of day 3 during the on-column cleavage, equilibrate the HiLoad 16/600 Superdex 200 column with one round of washing using one column volume (120 mL) of dH_2_O, followed by an additional wash with one column volume of the gel filtration buffer at 4°C.

### Purify, concentrate, and store recombinant BAX

Day 4: Load 20 mL of the prechilled lysis buffer onto the chitin column and pipette the chitin resin up and down several times to unpack the beads. Unseal the chitin column and collect the entire eluate (approximately 22–23 mL). Note: it is strongly recommended that the investigators reserve a 100-μL aliquot of the eluate to evaluate the efficacy of the on-column cleavage (“Chitin Elute;” Figure 2A). To further purify and isolate monomeric BAX, load an appropriate volume of the eluate onto the equilibrated HiLoad 16/600 Superdex 200 column according to the manufacturer’s instructions. The ÄKTA pure™ 25 system parameters are set as follows to conduct the chromatography at 4°C: sample volume = 5 mL; system flow rate = 1 mL/min; fraction = 2 mL; alarm system pressure = 0.3 MPa. Perform the chromatography as many times as necessary to fully use the entire eluate.

To identify the BAX-containing fractions and determine the purification efficiency, take 40 μL from alternating fractions, add the Laemmli sample buffer, and subject aliquots to gel electrophoresis (i.e., SDS-PAGE). Stain protein gels with the GelCode™ Blue Stain Reagent (or Coomassie equivalent) according to the manufacturer’s instructions to visualize the protein bands (see Figure 2D). Pool the fractions containing only monomeric BAX and combine it with fractions from each round of chromatography. Concentrate protein to a volume of approximately 3 mL by centrifuging in an Amicon® Ultra-4 Centrifugal Filter at 4°C according to the manufacturer’s instructions. Thoroughly mix the resulting 3 mL BAX sample with a pipette and quantify the BAX concentration using the Pierce™ BCA Protein Assay Kit. Based on the protein concentration and sample size volume, calculate the total yield of recombinant BAX and continue to concentrate BAX to an ideal concentration range of 30–40 μM using the same Amicon^®^ Ultra-4 Centrifugal Filter (note: BAX may bind to the filter membrane, and using the same filter may help to avoid further loss of the BAX protein). Aliquot the recombinant BAX into single-use aliquots (e.g., 20 μL per tube or suitable volume for specific downstream applications), flash-freeze using liquid nitrogen, and store at −80°C for future experimental interrogation.

### Large unilamellar vesicle permeabilization assays (optional)

Large unilamellar vesicles (LUVs) are prepared as previously described (Kuwana et al., 2002; Asciolla et al., 2012). Briefly, phosphatidylcholine, phosphatidylethanolamine, phosphatidylserine, phosphatidylinositol, and cardiolipin are combined at a ratio of 47:28:9:9:7 (5 mg total, for 7% cardiolipin LUVs) or 48:28:10:10:4 (5 mg total, for 4% cardiolipin LUVs), respectively; dried under a nitrogen gas environment; and resuspended in LUV buffer containing a polyanionic dye (12.5 mM ANTS: 8-aminonaphthalene- 1,3,6-trisulfonic acid) and cationic quencher (45 mM DPX: *p*-xylene-bis-pyridinium bromide) using a water bath sonicator. Unilamellar vesicles are formed by extrusion of the suspension through a 1.0-μm polycarbonate membrane. The unincorporated DPX and ANTS are removed by using a 10-mL Sepharose CL-2B gravity flow column. LUV preparations are only used for <2 weeks from when they are generated to avoid significant liposome degradation. For LUV permeabilization assays, we use a 96-well format and 100 μL of the total volume per condition; BAX, BIM^BH3^, C8-BID, and buffers are combined as indicated and analyzed for fluorescence (excitation wavelength: 355 nm; emission wavelength: 520 nm) by using a multi-mode microplate reader. The percentage of release is calculated between the baseline provided by the buffer control and 100% release obtained by LUVs solubilized in 1% CHAPS.

### Mitochondrial outer membrane permeabilization assays (optional)

Mitochondria isolation and MOMP assays are performed as previously described (Renault et al., 2013). In brief, for the MOMP assays, 50 μg of mitochondria is treated with BAX, C8-BID, BIM^BH3^ peptide, and buffers as indicated and incubated in TIB supplemented with 100 mM KCl at 30°C for 1 h. Every assay contains a 0.1% Triton X-100 control to determine maximal cytochrome *c* release. The samples are then centrifuged at 5,500 × *g* for 10 min to separate them into pellets and supernatants. Equal percentages of the resulting pellet and supernatant fractions are analyzed by SDS-PAGE and Western blot to assess cytochrome *c* release.

### Kinetic fluorescence polarization assays (optional)

Fluorescence polarization assays for monitoring BAX early activation (FLAMBE) are performed as previously described (Gelles et al., 2022; Mohammed et al., 2022). Briefly, by using a 96-well format and 100 μL of the total volume per condition, BAX, BIM^BH3^, C8-BID, buffers, and fluorescently labeled BAK^BH3^ peptide (BAK^TAMRA^) are combined as indicated and analyzed for fluorescence polarization (excitation wavelength: 530 nm; emission wavelength: 590 nm) using a multi-mode microplate reader fit with a polarization filter.

## Anticipated results

### Systematic evaluation of BAX protein expression and purification

During this workflow, investigators should systematically collect the protein samples at each key step to confirm expression, capture, and purification of monomeric BAX (highlighted in the Methods). When using the pTYB1–BAX construct, the IPTG-induced intein-CBD-tagged BAX protein band is detected at 75 kDa (Figure 2A; first two lanes). Following three rounds of passing the bacterial lysate through the chitin column, the chitin column effectively captures the majority of recombinant BAX (Figure 2A; third and fourth lane). Investigators should also confirm the release of BAX from the chitin column following the DTT-induced intein cleavage by sampling the eluate. As anticipated, the CBD was removed during the DTT-induced cleavage process and BAX from the chitin column eluate was detected at 21 kDa (Figure 2A; last lane). The eluate from the chitin column is not entirely devoid of contaminating species, as multiple higher molecular weight species were revealed through protein staining (Figure 2B; last lane), so investigators need to perform additional purification using size exclusion chromatography.

The size exclusion chromatography technique separates the contaminating species and other forms of BAX from monomeric BAX, allowing the purification of BAX in the monomeric form. Prior to carrying out chromatography, we suggest that the investigators determine the resolution of their columns based on the manufacturer’s manual or run molecular standards. Following size exclusion chromatography, the chitin column eluate should be fractionated into two distinct peaks. The first peak contains higher molecular weight species, and the second peak is a BAX monomer peak (Figure 2C). Then, investigators need to identify fractions containing monomeric BAX within the second peak and evaluate their purity by performing protein staining on alternating fractions from the size exclusion chromatography. Due to its relatively small size (21 kDa), BAX is typically eluted in later fractions (e.g., fraction 44–52; Figure 2D); however, the specific fractions containing BAX vary depending on the type of chromatography column and collection volume.

In the initial protein purification, investigators need to perform a Western blot using the BAX antibody to confirm that the observed protein is BAX. BAX bands should be detected at the same size as observed in the protein staining analysis (Figure 2E). It is worth noting that BAX is able to dimerize during SDS-PAGE in a denaturing condition, so an additional BAX dimer band may be detected by the Western blot. However, the dimer band does not suggest the presence of a BAX dimer in these fractions (e.g., 44–52), as suggested by the molecular standard (Figure 2C). Since a Western blot has improved detection, it is expected that BAX can be detected in early fractions (e.g., fraction 24–28), suggesting the presence of BAX higher molecular-weight species in the chitin column eluate. Collectively, fractions 44–52 were confirmed to exclusively contain monomeric BAX and pooled for concentration and storage. Fractions 54–56 also exclusively contain monomeric BAX, but the total amount together is too little to improve the yield. Moreover, pooling fractions 54–56 for concentrating significantly prolongs the amount of time spent on concentrating BAX, so we recommend not collecting fractions 54–56. Typically, 2 L of the bacterial culture generates 2 mL of 30 μM (∼1.4 mg) recombinant BAX. If investigators are concerned about the purity of their BAX, we recommend running a sample of the concentrated stock on a gel and detecting with GelCode or a similar reagent, which would confirm both a lack of contaminating species and a lack of multimeric BAX populations (Figure 2F). That being said, the size exclusion chromatography step should eliminate contaminating proteins and multimeric BAX, so investigators likely do not need to confirm it with additional techniques.

### Evaluate the function of recombinant BAX with the large unilamellar vesicle permeabilization assay

**FIGURE 3:**
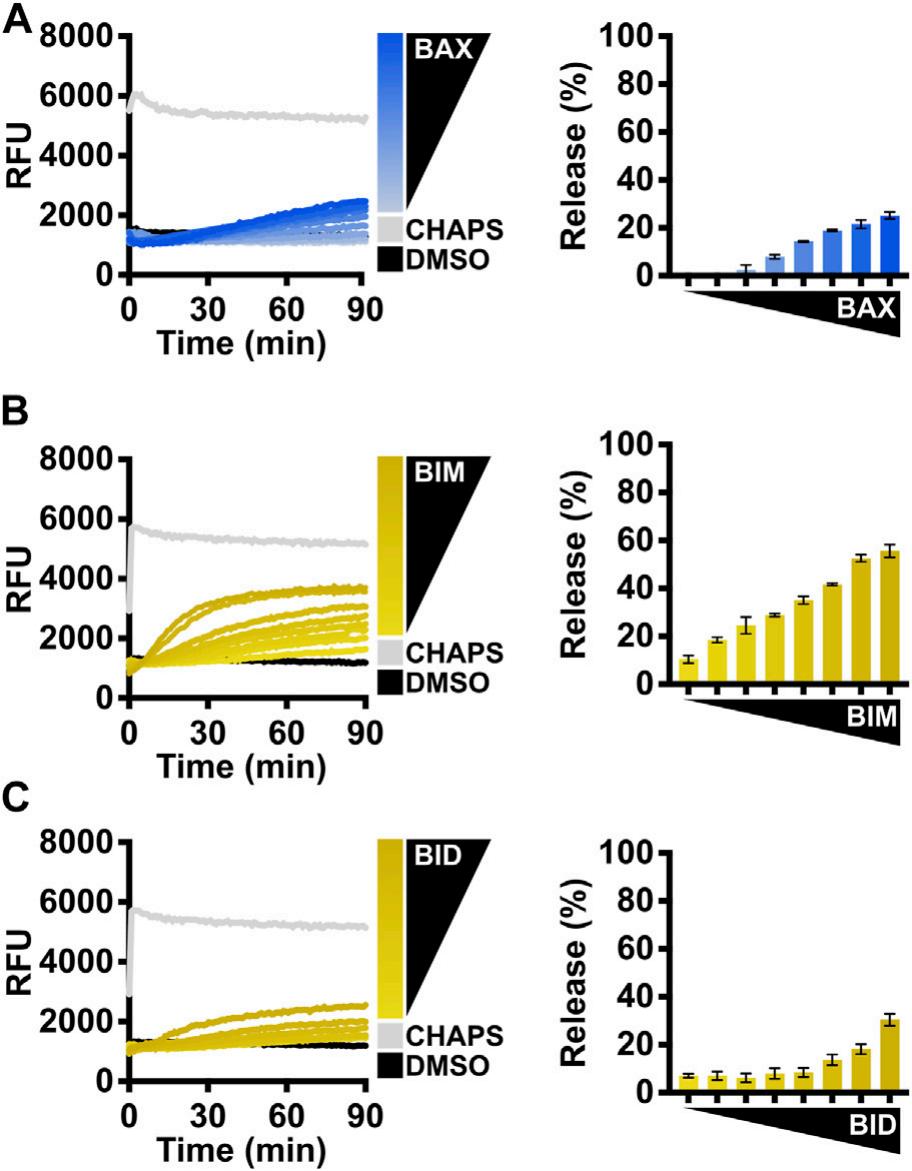
Evaluate the BAX function with BCL-2 family direct activators. **(A)** Left: kinetics of LUVs permeabilized by concentration-dependent auto-activated BAX [105–500 nM]; right: normalized endpoint of LUV permeabilization. **(B)** Left: kinetics of LUVs permeabilized by BAX [160 nM] with the presence of BIM^BH3^ [1:0–1:25 M excess], demonstrating that recombinant BAX is activated by BIM; right: normalized endpoint of LUV permeabilization. **(C)** Left: kinetics of LUVs permeabilized by BAX [160 nM] with the presence of BID^BH3^ [1:0–1:75 M excess], demonstrating that recombinant BAX is activated by BID; right: normalized endpoint of LUV permeabilization. Kinetic data are represented as the mean of triplicates; error bars denote SD.

The apoptotic function of BAX is to form proteolipid pores on the OMM and induce MOMP. To evaluate the function of recombinant BAX, the investigators need to conduct a series of membrane permeabilization assays using model membranes and/or isolated mitochondria. Large unilamellar vesicles (LUVs) are biochemically defined liposomes that mimic the composition of the OMM and are well-suited for testing BAX function (Kuwana et al., 2002). Here, we present a series of different settings for investigators to evaluate the function of BAX using LUV permeabilization assays. The first experiment tests a range of BAX concentrations (e.g., 100–500 nM) for LUV permeabilization (Figure 3A). This experiment serves two purposes: 1) the BAX protein exhibits concentration-dependent auto-activation, and permeabilization should be observed at higher concentrations; 2) researchers can identify a suitable non-activating concentration of BAX for subsequent experiments. The BAX titration experiment will demonstrate auto-activation but not necessarily activation of the entire BAX population or the maximal amount of LUV permeabilization for that concentration of BAX. Therefore, investigators should test BAX activation using either BCL-2 family direct activators (e.g., BID and BIM) or activating detergents.

#### BCL-2 direct activator-mediated BAX activation

The activity of BAX is primarily regulated by its interaction with other BCL-2 family proteins, so we recommend investigators conduct LUV permeabilization assays with different forms of BCL-2 direct activators for BAX. In cells, BIM is the predominant activator of BAX (Sarosiek et al., 2013), so we suggest investigators first test BAX treated with a titration of the BIM^BH3^ peptide (Figure 3B). This experiment serves two purposes: 1) the BIM^BH3^ peptide exhibits a concentration-dependent activation on the BAX membrane permeabilization function, confirming that BAX responds to BH3 stimulation; 2) investigators can identify a suitable molecular ratio for future experiments (e.g., synergistic/antagonistic test with a BAX activity modulator).

Another direct activator, BID, typically activates BAX following death receptor signaling (Li et al., 1998), so the investigators may choose the BID^BH3^ peptide if their primary interest is to study BID-mediated BAX activation, albeit it requires a much higher concentration than the BIM^BH3^ peptide (Figure 3C). The potency of the BH3 peptide largely depends on the length of the peptide (Gelles et al., 2022). The commercially available BID^BH3^ peptide is six amino acids shorter than the BIM^BH3^ peptide, so we anticipate that BID^BH3^ would show decreased potency in activating BAX.

**FIGURE 4:**
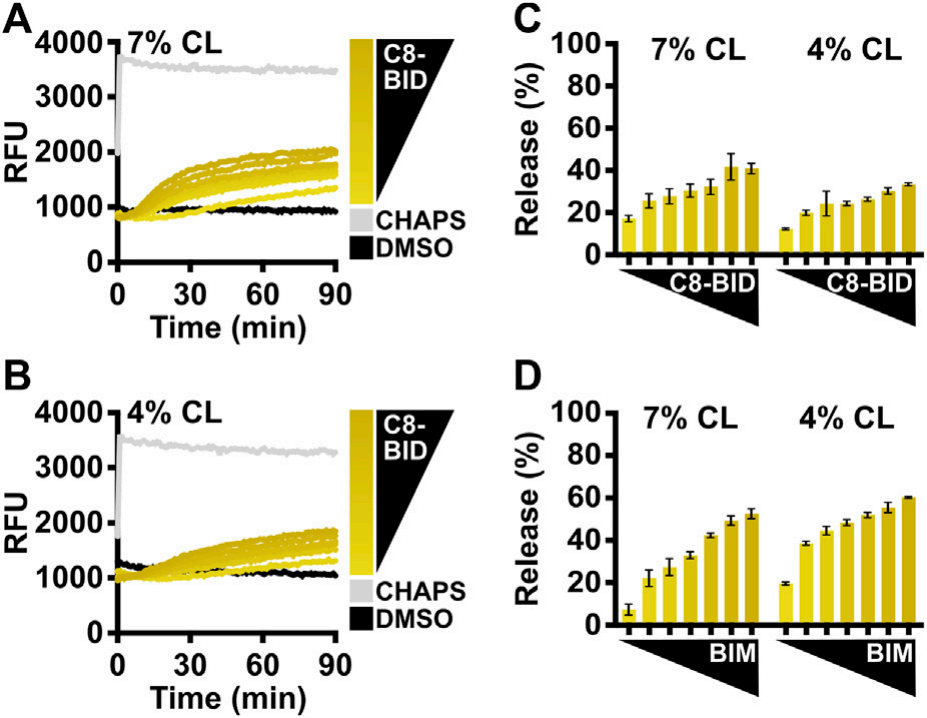
Comparison of BCL-2 direct activators using LUVs with different cardiolipin percentages. **(A)** Kinetics of LUVs permeabilized by BAX [160 nM] in the presence of C8-BID [33–250 nM] using 7% cardiolipin (“CL”) LUVs, demonstrating that recombinant BAX is activated by C8-BID. **(B)** Kinetics of LUVs permeabilized by BAX [160 nM] in the presence of C8-BID [33–250 nM] using 4% cardiolipin (“CL”) LUVs. **(C)** Normalized endpoint of LUV permeabilization from **(A,B)**. **(D)** Normalized endpoint of LUVs permeabilized by BAX [160 nM] activated by BIM^BH3^ [1:0–1:25 M excess] using 7% or 4% cardiolipin (“CL”) LUVs. Kinetic data are represented as the mean of triplicates; error bars denote SD.

The caspase-8 cleaved BID (C8-BID) protein has been historically utilized as a major *in vitro* direct activator for BAX, so investigators may alternatively use C8-BID to study BAX activation. C8-BID shows increased potency in activating BAX at nanomolar range due to its tertiary structure (Figure 4A). Several studies suggest that cardiolipin recruits C8-BID to the OMM and promotes the function of cleaved BID to activate BAX (Lutter et al., 2000; Kuwana et al., 2002). Therefore, it is anticipated that C8-BID exhibits greater synergy with BAX when using LUVs composed of a higher percentage of cardiolipin (i.e., 7%), resulting in a higher amount of membrane permeabilization at an increased rate (Figures 4A–C). For investigators primarily using BIM to study BAX activation, there is no notable difference between using LUVs with 7% cardiolipin and those with 4% cardiolipin (Figure 4D).

#### Detergent-mediated BAX activation

**FIGURE 5:**
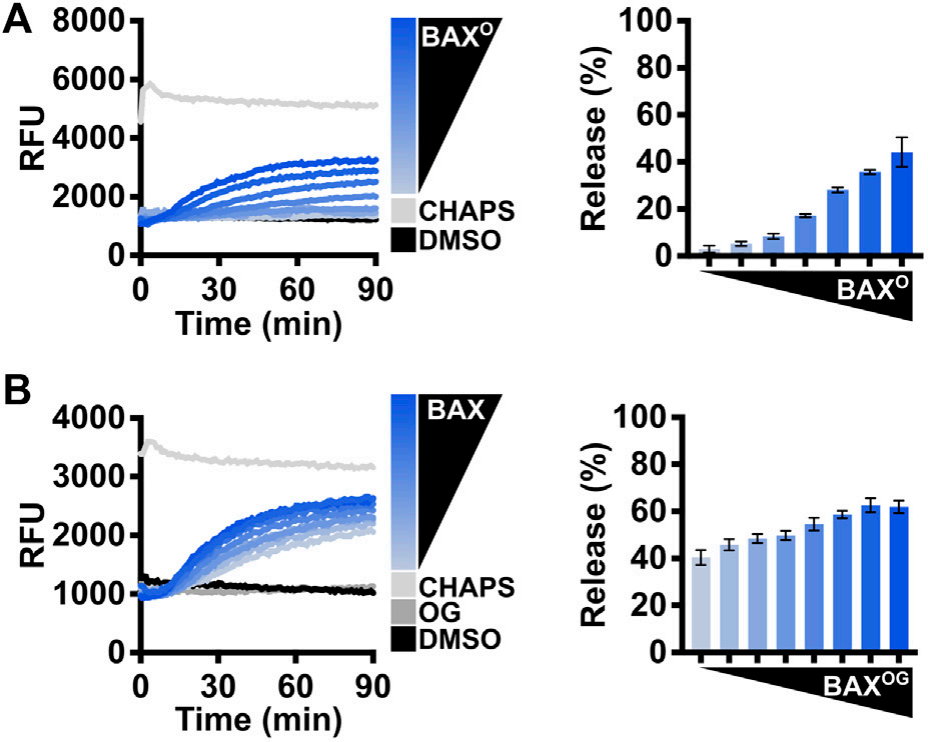
Evaluate the BAX function with detergents. **(A)** Left: kinetics of LUVs permeabilized by DDPC-induced BAX oligomers [“BAX^O^;” (44–500 nM)]; right: normalized endpoint of LUV permeabilization by BAX^O^. BAX^O^ was produced by incubating BAX with DDPC [1 mM] for 14–16 h at 4°C. **(B)** Left: kinetics of LUVs permeabilized by OG-activated BAX [OG: 0.2%; BAX: 105–500 nM]; right: normalized endpoint of LUV permeabilization by OG-activated BAX. Kinetic data are represented as the mean of triplicates; error bars denote SD.

Investigators can also use *n*-dodecyl-phosphocholine (DDPC), a detergent that has been reported to create homogenous BAX oligomers mimicking physiological activation (Hauseman et al., 2020), to assess the enhanced LUV permeabilization for several concentrations of BAX (Figure 5A). Alternatively, the detergent octyl-β-glucoside (OG) has been long-established for activating BAX (Hsu and Youle, 1998) and may be used for assessing real-time BAX activation and LUV permeabilization at several concentrations of BAX (Figure 5B). Collectively, the aim of performing these assays is to demonstrate that the BAX protein is functional and to determine an appropriate concentration of BAX that will not auto-activate but will robustly permeabilize LUVs and exhibit an appropriate dynamic range for subsequent experiments.

Collectively, these results are fairly reproducible, so if a batch of the BAX proteins deviates from the expected results, it may suggest issues with the protein quality, purity, or concentration.

### Evaluate the BAX function with isolated mitochondria

**FIGURE 6:**
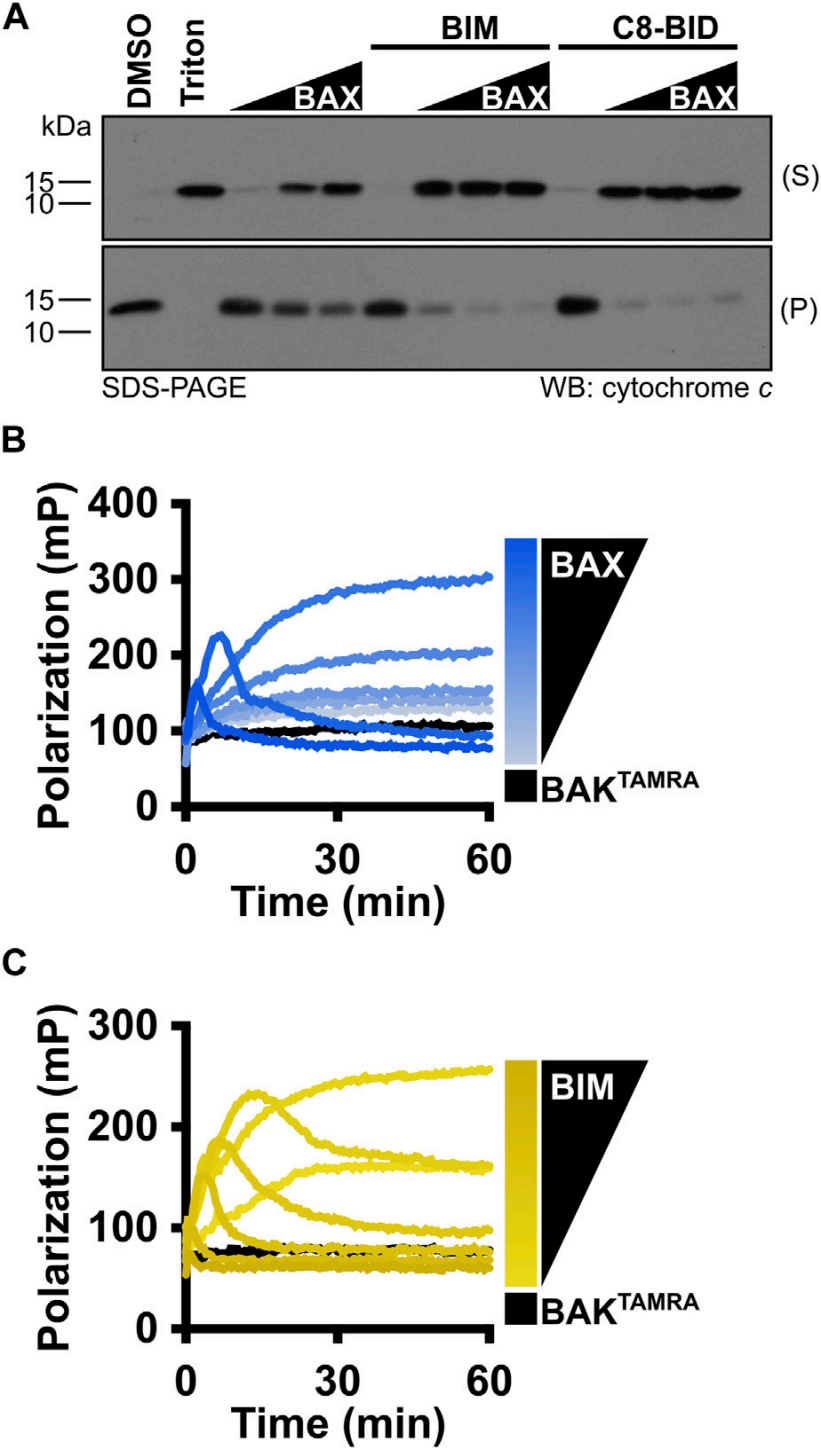
MOMP and FLAMBE assays are valuable techniques to study BAX activation. **(A)** Isolated mitochondria were incubated with BAX [5, 25, and 50 nM], C8-BID [25 nM], and the BIM^BH3^ [2.5 μM] peptide, as indicated. Mitochondria were centrifuged, and equal percentages of the resulting supernatant (S) and pellet (P) were subjected to Western blot detection of cytochrome *c*. **(B)** BAK^TAMRA^ [50 nM] was added to BAX [17–200 nM], and fluorescence polarization was measured. **(C)** BAK^TAMRA^ [50 nM] was added to BAX [60 nM] in the presence of BIM^BH3^ [0.26–2 μM], and fluorescence polarization was measured. Kinetic data are represented as the mean of replicates.

In cells, following BAX-mediated MOMP, cytochrome *c* is released into the cytosol to trigger caspase activation. Therefore, isolated mitochondria have been used as a fundamental standard to assess cytochrome *c* release for studying the BCL-2 family protein function. Mitochondria should be freshly isolated from *BAX* ^*−/−*^ *BAK* ^*−/−*^ double knockout MEFs and subject to MOMP on the same day to avoid cytochrome *c* leakage. Similar to LUV permeabilization assays, the investigators first need to test a range of BAX concentrations [10–50 nM], and BAX should activate in a concentration-dependent manner to permeabilize mitochondria and result in cytochrome *c* release into the supernatant (Figure 6A). This experiment serves to identify a suitable BAX concentration for subsequent BAX activation studies. Investigators then need to use the identified BAX concentration to recapitulate the synergy between BAX and the BIM^BH3^ peptide or C8-BID protein to establish an appropriate molecular ratio for their future research (Figure 6A).

### Monitor monomeric BAX activation with FLAMBE assays

Before BAX is fully competent to induce membrane permeabilization, it must undergo a series of conformational changes in the cytosol and OMM. For investigators who want to gain insight into the BAX early-activation process, we recommend using the FLAMBE assay (Mohammed et al., 2022). By using the fluorescently labeled BAK^BH3^ peptide capable of binding to the BAX BC groove, FLAMBE measures the activation-induced release of the BAK^BH3^ peptide to monitor BAX intramolecular conformational changes in BAX early activation. It can detect both concentration-dependent BAX auto-activation and BH3-stimulated BAX activation, indicated by a decrease in polarization signals (Figures 6B, C). Collectively, these assays interrogate BAX activation from different perspectives and corroborate with LUV permeabilization assays to appreciate BAX responses.

### BAX handling and storage

**FIGURE 7:**
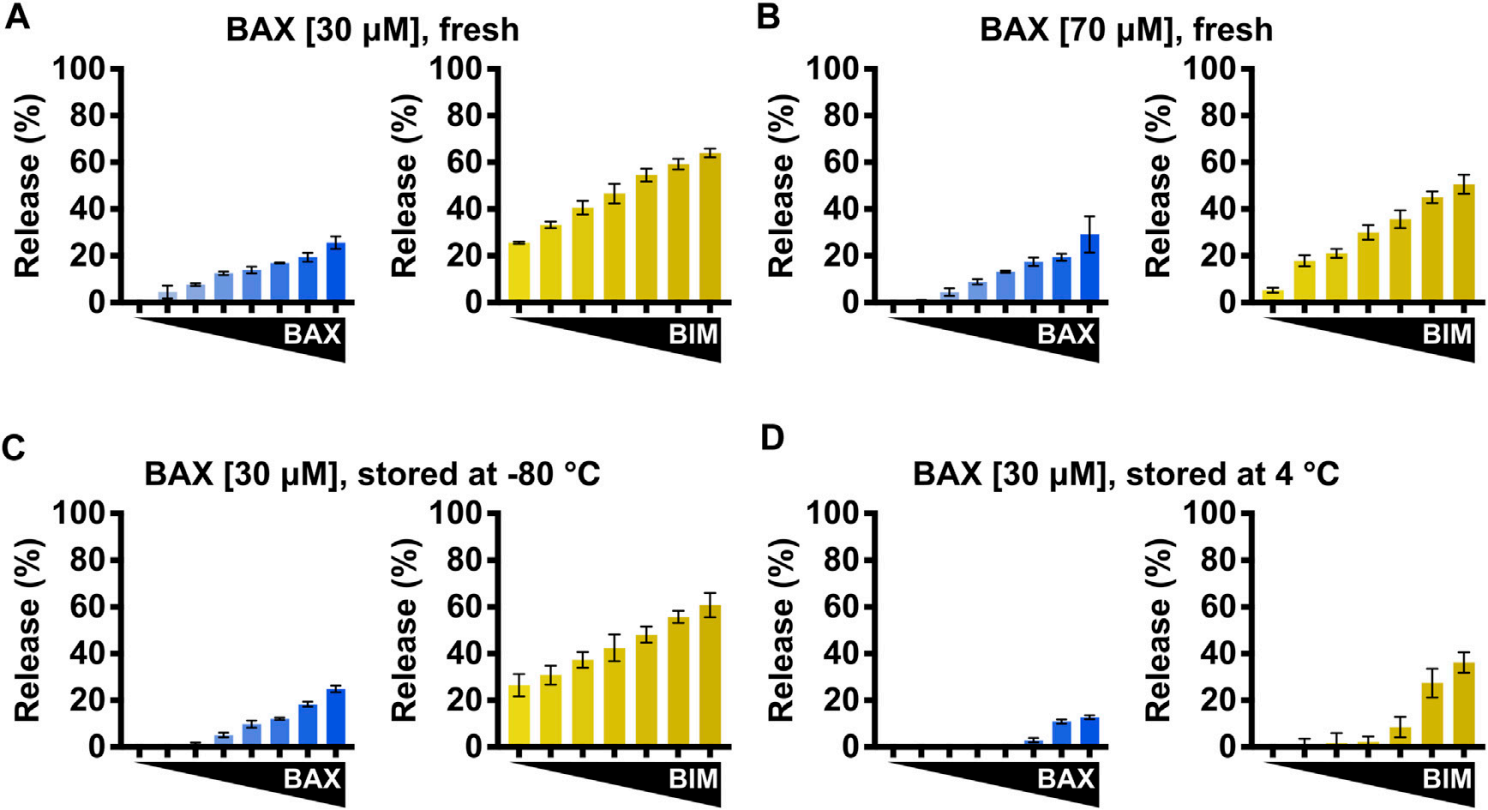
BAX storage conditions impact **BAX** function. **(A–D)** Left: normalized endpoint LUV permeabilization by auto-activated BAX [105–500 nM]; right: normalized endpoint LUV permeabilization by BIM^BH3^-activated BAX [160 nM] in the presence of BIM^BH3^ [1:0–1:25 M excess]. **(A)** Purified BAX was concentrated to 30 μM and immediately subjected to the LUV assay. **(B)** Purified BAX was concentrated to 70 μM and immediately subjected to the LUV assay. **(C)** Purified BAX was concentrated to 30 μM and stored for 1 week at −80°C before being subjected to the LUV assay. **(D)** Purified BAX was concentrated to 30 μM and stored for 1 week at 4°C before being subjected to the LUV assay. Error bars denote SD.

Given the function of BAX, protein stocks tend to form multimers and aggregates during storage. Therefore, proper handling and storing of BAX stocks is key to preserving a functional and responsive BAX population for downstream investigations and consistent experimental results. We recommend concentrating BAX to a range of 30–40 μM since overconcentrating BAX can result in protein aggregation and, subsequently, a population that is less sensitive to BH3 stimulation (Figures 7A, B). Additionally, we observed BAX dimerization at high concentrations (data not shown), which may interfere with certain analyses (e.g., NMR and ITC). NMR and ITC analyses usually require higher protein concentrations to generate adequate signals, and typically 40 μM stocks are sufficient for these techniques. To store BAX, we recommend investigators aliquot BAX into single-use tubes (e.g., 10–20 μL for plate-based assays), flash-freeze them with liquid nitrogen, and store them at −80°C for future use. Investigators need to note that larger aliquots can slow the flash freezing, resulting in protein precipitation and loss. BAX stored at −80°C only exhibits a minor reduction in the BAX membrane-permeabilization activity but maintains its response to BH3 stimulation (Figure 7C). In contrast, BAX stored at 4°C exhibits a notable loss in BAX function at the same concentration and a reduced response to BH3 stimulation (Figure 7D). In conclusion, we suggest that researchers concentrate BAX to a range of 30–40 μM, aliquot it in small volumes, flash-freeze with liquid nitrogen, and store at −80°C for future use.

## Discussion

The mitochondrial pathway of apoptosis is fundamental for tissue homeostasis, and dysregulated control of this pathway is implicated in various human diseases including cancer, heart failure, and neurodegenerative disorders (Spitz and Gavathiotis, 2022). Given the critical role of BCL-2 family proteins in the regulation of apoptosis, research has been dedicated to developing small molecules to inhibit anti-apoptotic BCL-2 proteins (i.e., BH3 mimetics). Recent mechanistic studies of BAX molecular regulation have identified several BAX regulatory regions, providing key structural insights that aid in the development of small molecules capable of directly targeting BAX and modulating its activity (Gavathiotis et al., 2012; Pritz et al., 2017; Garner et al., 2019). These efforts were built upon the ability to generate recombinant BAX for structure–function interrogations and small-molecule screening.

Despite the increasing number of publications using recombinant BAX, the techniques listed in the Methods section are not sufficiently informative to learn BAX purification *de novo,* and there remains a lack of detailed and instructional protocols aimed at investigators who are not already familiar with the process of generating BAX protein. In this work, we present a comprehensive protocol using the pTYB1–BAX construct for expressing, purifying, and storing functional recombinant BAX with a yield of ∼1.4 mg per 2 L of bacterial culture. The resulting protein is properly folded, functional, and devoid of exogenous residues. To ensure the quality of each batch of BAX, we have recommended and provided examples of quality control and BAX activation assays. Additionally, given the high purity level of recombinant BAX, our two-step chromatography protocol can be easily adapted to generate N^15^- or C^13^-labeled BAX for HSQC NMR studies (data not shown).

Moreover, while this protocol is capable of generating wild-type full-length BAX, it is also suitable for purification of BAX mutants, following mutagenesis of the expression vector. It is worth noting that some structural mutants of BAX are created by introducing cysteine residues to form disulfide bonds, and these BAX mutants must be oxidized by incubating them in an oxidative environment to generate the disulfide tether (Gavathiotis et al., 2010; Czabotar et al., 2013; Gelles et al., 2022). While not being physiological activators, both DDPC and OG are beneficial for evaluating loss-of-function BAX mutants as they may bypass certain structural requirements for BAX oligomerization and serve as robust positive controls for BAX activity.

Recently, a high-yield protocol was developed to generate recombinant BAX for biophysical interrogations (Dingeldein et al., 2019). This protocol uses a double-fusion construct with an N-terminal GST tag and a C-terminal intein–CBD tag to prevent BAX membrane insertion and bacterial cytotoxicity. While this protocol has a yield of 1–2 mg per liter of bacterial culture, it lacks a dedicated technique to isolate monomeric BAX. Additionally, their purification strategy requires protease treatment, multiple buffer exchange steps, and extends for over 6 days. By comparison, our protocol comprises a convenient 4-day timeline with straightforward operations and achieves 0.7–0.8 mg per liter of bacterial culture—a suitable yield for extensive biochemical and biophysical investigations. Importantly, our protocol uses a size exclusion chromatography step to ensure the purification of monomeric BAX. While the Dingeldein protocol may be appropriate for investigators who require a large quantity of recombinant BAX, our protocol is well-suited and streamlined for investigators interested in BAX functional studies consistent with a significant literature.

Collectively, we optimized the handling and storage conditions for BAX, and BAX can be stored at −80°C for up to 6 months with consistent and reproducible function. We recommend a storage concentration of 30–40 μM to avoid BAX multimerization, and this range is suitable for most techniques essential for BAX research.

## Data Availability

The original contributions presented in the study are included in the article/Supplementary Material; further inquiries can be directed to the corresponding author.
